# Low-Vacuum Deposition of Glutamic Acid and Pyroglutamic Acid: A Facile Methodology for Depositing Organic Materials beyond Amino Acids

**DOI:** 10.1155/2014/434056

**Published:** 2014-09-01

**Authors:** Iwao Sugimoto, Shunsaku Maeda, Yoriko Suda, Kenji Makihara, Kazuhiko Takahashi

**Affiliations:** ^1^School of Computer Science, Tokyo University of Technology, Katakura, Hachioji, Tokyo 192-0982, Japan; ^2^Graduate School of Bionics, Tokyo University of Technology, Katakura, Hachioji, Tokyo 192-0982, Japan; ^3^Shiseido Co., Ltd., Higashi-shimbashi, Minato-ku, Tokyo 105-8310, Japan; ^4^Bionanotechnology Center, Tokyo University of Technology, Katakura, Hachioji, Tokyo 192-0982, Japan; ^5^Faculty of Science and Engineering, Doshisha University, Kyotanabe, Kyoto 610-0321, Japan

## Abstract

Thin layers of pyroglutamic acid (Pygl) have been deposited by thermal evaporation of the molten L-glutamic acid (L-Glu) through intramolecular lactamization. This deposition was carried out with the versatile handmade low-vacuum coater, which was simply composed of a soldering iron placed in a vacuum degassing resin chamber evacuated by an oil-free diaphragm pump. Molecular structural analyses have revealed that thin solid film evaporated from the molten L-Glu is mainly composed of L-Pygl due to intramolecular lactamization. The major component of the L-Pygl was in *β*-phase and the minor component was in *γ*-phase, which would have been generated from partial racemization to DL-Pygl. Electron microscopy revealed that the L-Glu-evaporated film generally consisted of the 20 nm particulates of Pygl, which contained a periodic pattern spacing of 0.2 nm intervals indicating the formation of the single-molecular interval of the crystallized molecular networks. The DL-Pygl-evaporated film was composed of the original DL-Pygl preserving its crystal structures. This methodology is promising for depositing a wide range of the evaporable organic materials beyond amino acids. The quartz crystal resonator coated with the L-Glu-evaporated film exhibited the pressure-sensing capability based on the adsorption-desorption of the surrounding gas at the film surface.

## 1. Introduction

L-glutamic acid (L-Glu) has been widely investigated due to its greatly beneficial properties in the food and pharmaceutical industries where its polymorphism and crystalline shape have received considerable attention [1, 2]. It should be noted that the transformation between the metastable *α* form of L-Glu and its stable *β* form has been widely studied not only out of scientific interest, but also from the application aspect [3–5].

The polymorphs of amino acids are ascribed to the complex interactions between the hydrogen bonding moieties (amino and carboxy groups) and side chains, which permit the steric and electrostatic interactions that specifically form the well-ordered aggregates in the solid phase. The L-Glu polymorph is substantially governed by the degree of charge separation in the main interaction moieties of the amino and carboxy groups, whose charging state is either zwitterionic or neutral [6]. Because the molecular packing arrangements of two polymorphs (*α* and *β* forms) of L-Glu differ significantly, the transformation between polymorphs in the solid state would hardly occur at room temperature [7].

The thermal effects on the polymorphs of L-Glu have been an intriguing subject in solid-phase biochemistry. In previous studies, the molten *α* form of L-Glu was eventually transformed into polyglutamic acid via stepwise changes of a *β* form of L-Glu followed by a pyroglutamic acid (Pygl) [8–11].

Amino acids are expected to provide the biofriendly thin layers, on which biological microorganisms and biological molecular devices would be adequately immobilized maintaining their activities. Moreover, amino acids possessing multifunctional groups are promising motif molecules for constructing the well-ordered molecular networks for potential electro-optical applications [12–14]. Thermal stability of amino acids plays an important role in depositing thin solid films of amino acids by means of evaporation-sublimation processes. To our knowledge, even though some groups have reported the preparation of amino acid films by vacuum deposition [15, 16], the amino acid films deposited by dry processing have not been well investigated mainly due to lack of industrial applicability.

The sublimation deposition of L-Glu, which is commonly used for biological application, has not yet been carried out successfully due to the thermal decomposition of L-Glu [6, 17, 18]. The main objective of this study is to develop the evaporation of molten L-Glu in a low-vacuum condition to form thin solid films via solidification at the substrate. The molten amino acid evaporated in low vacuum is considered to be more stable thermodynamically than the sublimation from solid state in high vacuum, due to small energy of the phase-shift from liquid to vapor. In this study, vacuum evaporation from the molten state was implemented by original low-vacuum coater, which is feasible for coating a wide range of organic materials that are evaporable under low-vacuum conditions.

**Figure 1:**
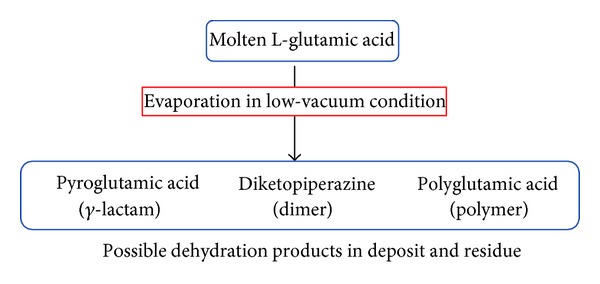
Schematics of possible changes to L-Glu during the course of evaporation.

During evaporation in low-vacuum condition, the molten L-Glu may induce dehydration reactions such as intramolecular cyclization, intermolecular dimerization, and polymerization as conceptually expressed in Figure 1. The thermally stable derivatives of L-Glu and Pygl are expected to form the thin layer of deposits characterized by the unique molecular networks induced by their multifunctional groups [19]. Transformation of polymorphs and rearrangement of molecular structure may occur as a result of thermal evaporation and deposition processes.

Thin amino acid films with the well-ordered molecular networks may be useful for not only biofriendly surface conditioning but also gas sensing based on the structurally specific intermolecular interactions.

## 2. Materials and Methods

### 2.1. Vacuum Evaporation

**Figure 2:**
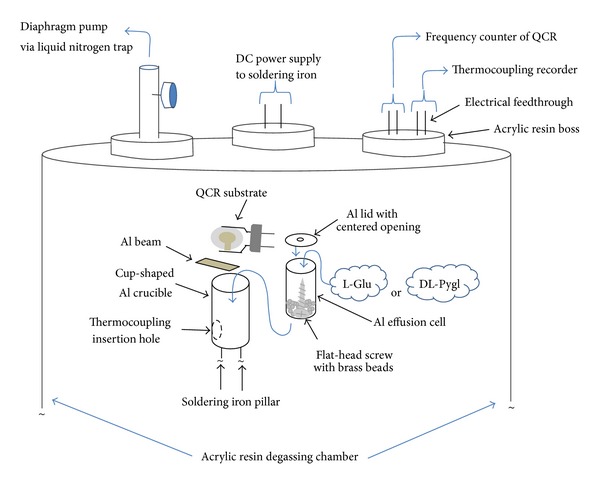
Schematics of the low-vacuum coater.

Our original low-vacuum coater was fabricated by a soldering iron (Goot, KS-60R), which stood straight on a metal mount screwed onto the base plate of the 12-liter acrylic-resin degassing chamber (As-one, VZ) based on our previous work [20]. A cup-shaped aluminum (Al) crucible with an Al lid with a 2 mm central opening was bolted to the top of the soldering iron, as shown in Figure 2.

The crucible was impregnated with a cup-shaped Al effusion cell (3 mL) charged with 450 mg evaporant powder. L-Glu (>99.0%, Wako) or DL-Pygl (>99%, Wako) was used as an evaporant, which was dusted over a flat-head steel screw surrounded by twenty-five 2 mm brass beads (ca. 0.92 g) placed in the effusion cell to ensure homogenous thermal conduction.

To prevent direct spitting to the deposition substrate, a 7 mm wide Al beam was inserted between the perforated lid and the substrate of quartz crystal resonator (QCR) separated by 25 mm. Without metal conductors in the effusion cell and the Al beam crossing the evaporant flow, the spitting of the molten L-Glu has notably formed the inhomogeneous granules in the deposited films. The evaporated materials were to transverse a roundabout path onto the substrate over the crossing beam. A glass plate or a polymer-coated metal grid was also used as a film substrate for structural analyses.

During steady evaporation, the crucible temperature was maintained at about 180°C with a DC power supply (Shimaden, DSM temperature controller unit) set at 50 V. Before heating, a background pressure of ca. 210 Pa was attained with a 20 L/min diaphragm pump (ULVAC, DAV-20) via a liquid nitrogen-cooled trap. The maximum pressure during evaporation was increased to ca. 380 Pa at about 180°C.

### 2.2. QCR Used for Deposition Monitoring

The deposition monitoring was carried out by using an AT-cut QCR (9 MHz) with Au electrodes, connected to a frequency counter (Q-pod, Inficon). The QCR surface was rugged without fine polishing. The electrode of QCR substrate was opposite to the opening of the Al lid of effusion cell. Prior to setting, the QCR substrate was cleaned by UV/ozone irradiation for 30 min with an excimer lamp (Ushio, UER20H-172), whose emission peak was centered at 172 nm with 2 W power. This UV/ozone cleaning has been also conducted for the other substrate, such as glass plate and Si wafer.

Based on Sauerbrey's equation [21], the loaded mass (Δ*m*) on the QCR can be correlated with the shift of resonant frequency (Δ*f*) as follows: Δ*m* [ng] = −1.05 Δ*f* [Hz]. Assuming that the evaporated materials are uniformly deposited on the QCR and their densities are 1.0 g/cm^3^, the corresponding film thickness (*t*) can be correlated with Δ*f *as follows:* t* [nm] = −54 Δ*f* [kHz]. Typical thickness of the L-Glu-evaporated film deposited on QCR was about 1000 nm, whereas that of the Pygl-evaporated film was about 2000 nm.

### 2.3. Structural Characterization

Scanning electron microscopy (SEM) images were obtained with a field emission scanning electron microscope (Jeol, JSM-7700F). The acceleration voltage and current of the primary electron beam were 5 kV and 5 *μ*A, respectively. The SEM sample was coated with Pt to prevent surface charging. Transmission electron microscopy (TEM) was performed with a field emission microscope (Jeol, JEM-3200FS) at 200 kV and 180 *μ*A. The TEM sample was directly deposited on a ^*ϕ*^80 *μ*m Cu grid coated with a 30-nm thick polyvinyl formal film (Jeol, No. 1606). Prior to sample deposition, the polyvinyl formal surface was hydrophilized by an ion sputtering in the residual air (Jeol, HDT-400).

The molecular structure of the film constituents was analyzed by ^1^H-NMR and ^13^C-NMR. The film deposited on a glass plate was shaved off with a sterilized scalpel. All of the deposited films were substantially dissolved in deuterium oxide (99.8 atom% deuterium) at a concentration of ca. 1 mM. The ^1^H-NMR and ^13^C-NMR spectra were recorded on a Bruker AV400 M spectrometer (Bruker BioSpin) with an UltraShield superconducting magnet system, in which the central magnetic field was 9.40 Tesla and magnetic energy was 28 kJ. The proton frequency was 400 MHz, and peak position calibration was carried out using the contaminated H_2_O as an inner standard at 4.79 ppm.

The films deposited on the gold QCR electrodes were analyzed using an infrared (IR) spectrometer (DIGILAB, FTS300) equipped with a microscope with ×150 objective magnification. The wavenumber resolution of the Hg-Cd-Te detector was 4 cm^−1^. Measurements for the evaporated films formed on the Au electrode of QCR were carried out by a reflection method. The transmission method was adapted for the KBr pellets of the original amino acids and the residues in the effusion cell after evaporation.

The powder X-ray diffraction (XRD) patterns were recorded on an X-ray diffractometer (Mac Science, MX Labo) using the films deposited on the dedicated glass plates. The excitation source was Cu K*α* (0.15418 nm, 40 kV, and 20 mA). The *θ*-2*θ* XRD spectra were obtained in a range of 10–100°, and the scan rate was 3°/min with intervals of 0.02°.

Thermal phase transformation of the films was analyzed by a differential scanning calorimeter (Shimadzu, DSC-60). The 2-mg sample shaved off the film deposited on glass substrate was packed in an Al pan covered with an Al lid. The temperature-increase rate was 5°C/min and the measurements were obtained under a 100 mL/min N_2_ flow.

## 3. Results and Discussion

### 3.1. Deposition Monitoring

**Figure 3:**
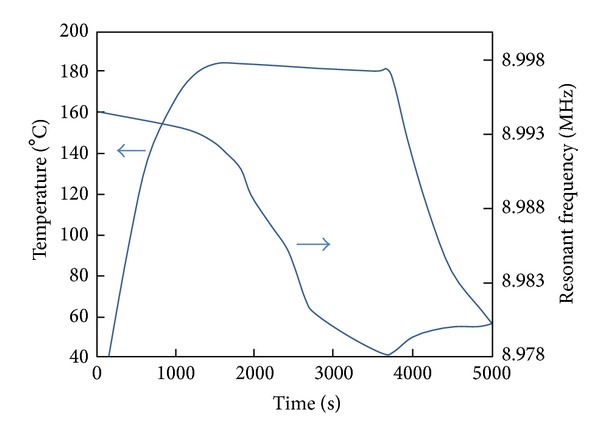
Typical time course of frequency shifts of QCR substrate and effusion cell temperature during L-Glu evaporation.

After evacuating to ca. 210 Pa, the crucible began to heat up with the application of a constant voltage of 50 V to the soldering iron.  Figure 3 shows the typical time course of frequency shifts of the QCR superimposed on the crucible temperature during L-Glu evaporation. Discontinuous abrupt shifts could be observed in the decreasing frequency-shift curves. These shifts are attributable to the massive loading of viscous deposits evaporated from the molten L-Glu [22, 23].

The frequency began to decrease notably at ca. 175°C indicating the consistent arrival of evaporated L-Glu on the QCR substrate. During evaporation of L-Glu, the chamber pressure increased above 250 Pa from 210 Pa at the background pressure before evaporation. The slight increase of resonant frequency after the termination of the heating is considered to be due to solidification of the viscous deposits by heat radiation.

### 3.2. Morphological Analyses of the Evaporated Films

**Figure 4:**
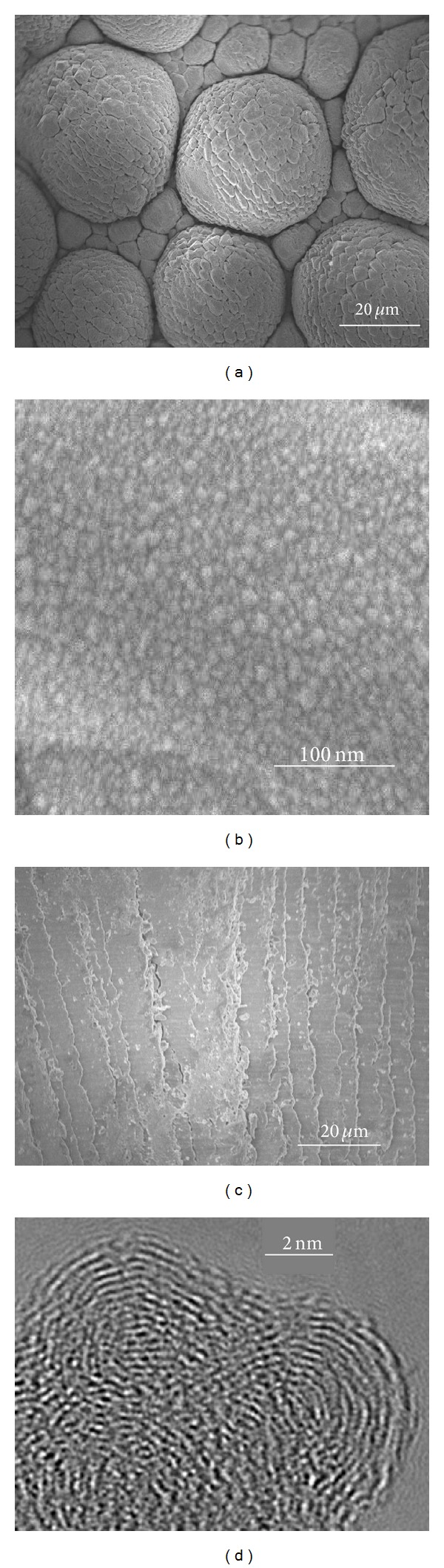
SEM images (a)–(c) of the deposits on Si wafer: (a) lower magnification of L-Glu-evaporated film, (b) higher magnification of L-Glu-evaporated film, (c) DL-Pygl-evaporated film, and (d) TEM image of L-Glu-evaporated film.

The L-Glu-evaporated film deposited on the glass plate was composed of closely packed polygonal blocks forming hemispherical domes, as shown in Figure 4(a). Its surface was covered with 20-nm particulates, as shown in Figure 4(b). The morphological characteristics mainly depended on the wettability of the viscous deposits.

We observed a series of wave lines on the surface of the DL-Pygl-evaporated film deposited on the glass plate, as shown in Figure 4(c). The DL-Pygl has been sublimated without a molten state, because the situation of residues in a crucible has not been changed after deposition. The sublimation deposition without molten state should not form the hemispherical domes as shown in the L-Glu film (Figure 4(a)).

High-resolution TEM images revealed the characteristic periodic patterns of the extremely thin deposit of the L-Glu evaporant, as shown in Figure 4(d). The semiconcentric periodic line spacing was ca. 0.2 nm, suggesting that the single-molecular interval of the crystallized molecular networks had been formed in the extremely thin deposit at the early stage of L-Glu evaporation.

### 3.3. Structural Characterization of the Evaporated Films

**Figure 5:**
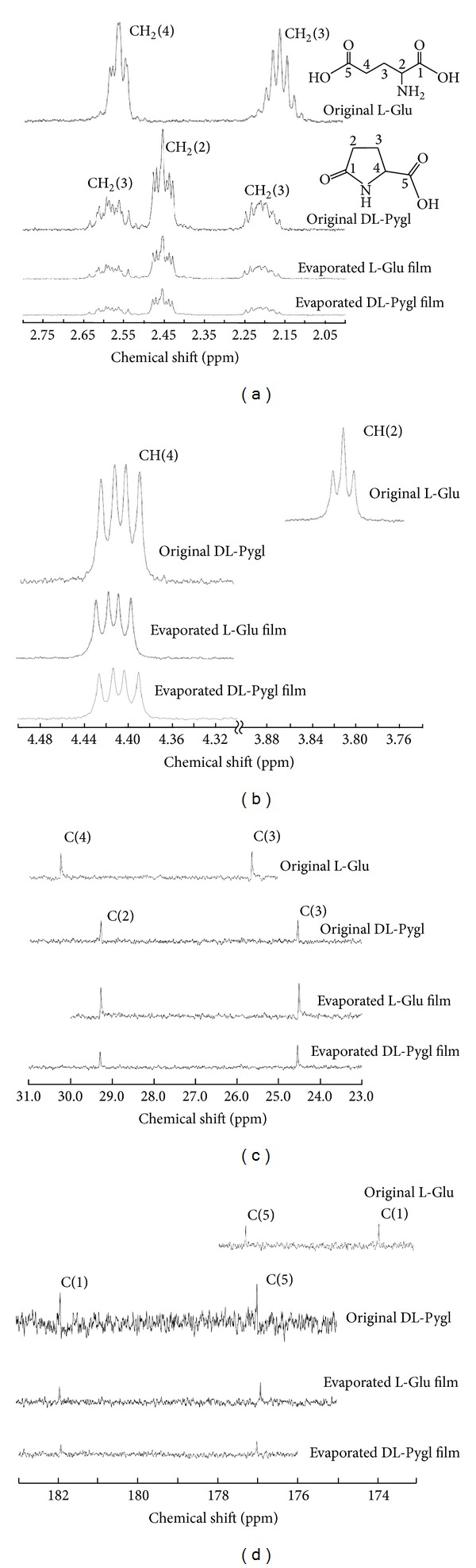
NMR spectra of evaporated films in comparison with their originals: (a) ^1^H-NMR spectra in highly shielded region, (b)^ 1^H-NMR spectra in moderately shielded region, (c)^ 13^C-NMR spectra in highly shielded region, and (d)  ^13^C-NMR spectra in slightly shielded region.

The ^1^H-NMR and ^13^C-NMR spectroscopic analyses clarified that the evaporated L-Glu film was exclusively composed of DL-Pygl [24, 25], as shown in Figures 5(a)–5(d). These NMR spectra are enough characterized to identify the molecular species, especially for the ^1^H-NMR spectra at high magnetic field as shown in Figure 5(a).

**Figure 6:**
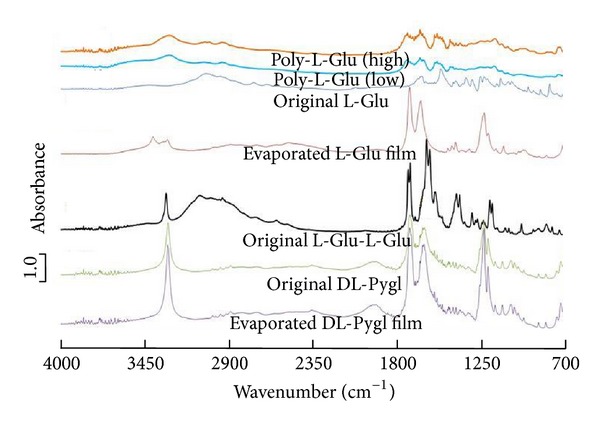
FTIR spectra of evaporated films recorded by the reflection-absorption method on QCR and their original powder measured by the KBr method.

Similar to the NMR analyses, FTIR analysis also revealed that the evaporated L-Glu film substantially consists of DL-Pygl, as shown in Figure 6. This molecular transformation from L-Glu to DL-Pygl is derived by thermal lactamization. Comparing to the original DL-Pygl, the evaporated L-Glu film exhibits the two weak peaks which appeared characteristically at about 3400 cm^−1^. These peaks are assignable to the N–H or O–H stretching bonds, which are expected to play important roles in making the molecular networks through hydrogen bonding. Similar to thermal lactamization of L-Glu to L-Pygl in solid state [8, 26], this lactamization of L-Glu presumably occurred over the course of evaporation of the molten L-Glu feasible to transform to L-Pygl through thermal intramolecular dehydration.

**Figure 7:**
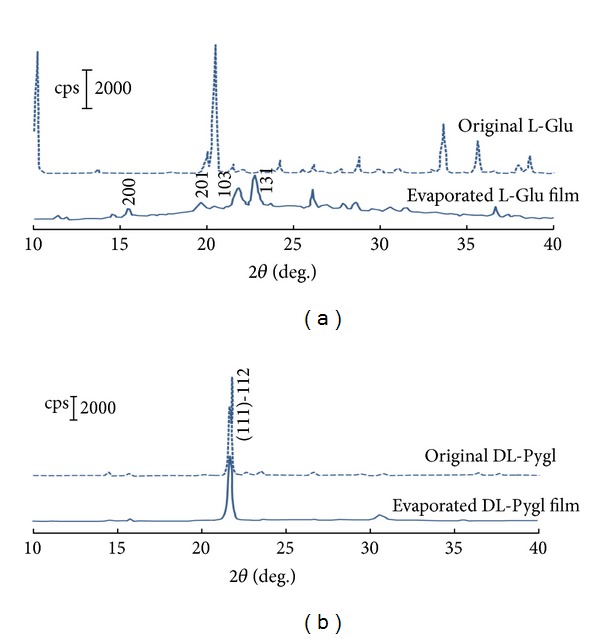
Powder XRD spectra of the evaporated films shaved off the glass substrate: (a) evaporated L-Glu film and (b) evaporated DL-Pygl film.

Irrespective of the peak intensities, the XRD patterns of the six evaporated L-Glu films were identical, suggesting that the crystalline polymorph had been constructed reproducibly. The typical XRD pattern of the evaporated L-Glu film is shown in Figure 7(a), which exhibits that the evaporated film is not composed of the original L-Glu. The main peak of the L-Glu-evaporated film was at ca. 22.7°, indicating that the film is constituted of L-Pygl, whose XRD pattern is expressed in Figure 7(b). The slight difference between two peaks is presumably due to the fact that the resulting L-Pygl is a mixture of the stable *β*-phase and the metastable *γ*-phase. The major component of the L-Pygl was in *β*-phase. And the L-Pygl contained slight amounts of *γ*-phase, which was presumably generated from the partial racemization to DL-Pygl [27]. In contrast, the DL-Pygl-evaporated film exhibited an exclusively strong peak, which was also observable in the original DL-Pygl at ca. 22.0°, as shown in Figure 7(b). This exclusive peak is attributable to the anisotropic effect of the highly oriented crystalline structure of the pristine DL-Pygl [19, 28].

**Figure 8:**
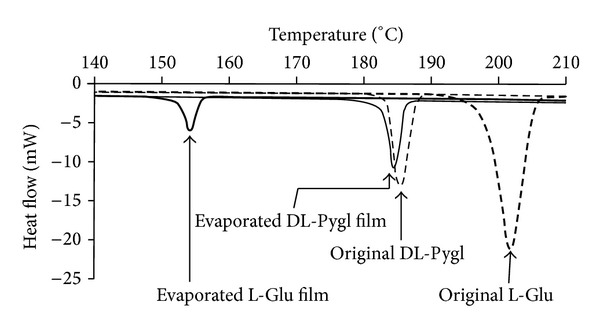
DSC curves of evaporated films and their original powders.

Differential scanning calorimetry (DSC) analyses revealed that the melting point of the L-Glu-evaporated film was at 154°C, whereas that of the original DL-Pygl was at 185°C, as shown in Figure 8. This original DL-Pygl melting point was almost the same as that of the DL-Pygl-evaporated film at 184°C. The cell temperature of steady evaporation ranged from 180°C to 185°C (as shown in Figure 3), which essentially coincides with the melting point of DL-Pygl at 185°C. These results suggest that the evaporants were generated after transformation from L-Glu to DL-Pygl by thermal intramolecular lactamization.

The eutectic melting of the mixture of the enantiomeric L-form and racemic DL-form should decrease the melting point of the L-Glu-evaporated film-constituting Pygl [27]. In the course of intramolecular lactamization of L-Glu, the resulting Pygl molecules partially induced racemization resulting in the DL-form. Based on the melting point of 154.4°C, we can estimate that the racemized DL-form is about several tens of % [28], in accordance with the aforementioned XRD analysis.

High-performance thin-layer chromatography (HPTLC) was carried out using a cellulose plate (Merck, 1.16092.0001) with a solvent mixture (1-butanol : acetone : water : acetic-acid = 20 : 20 : 46 : 14) as the mobile phase. For all the developed plates, we used 4 types of colorization reagents: Dragendorff reagent, molybdatophosphoric acid, ninhydrin, and iodine. We recognized that L-Pygl was the main component of the L-Glu-evaporated film, in which none of the original L-Glu remained. HPTLC also clarified that the L-Glu-evaporated film did not contain any type of peptide condensation derivatives, such as dimers, oligomers, or polymers.

XRD and DSC analyses revealed that the L-Glu-evaporated film was mainly made up of the *β*-phase of L-Pygl. Additionally, the film contained slight amounts of the *γ*-phase of L-Pygl, which was probably constructed from the partial racemization to DL-Pygl. Moreover, these analyses revealed that the DL-Pygl-evaporated film-constituting molecules retained not only molecular structure but also crystalline structure of the original DL-Pygl. This structural consistency between original source and the deposited film is attributable to deposition processes, which is conducted by sublimation without molten state.

### 3.4. Characterization of Residues in Effusion Cell

**Figure 9:**
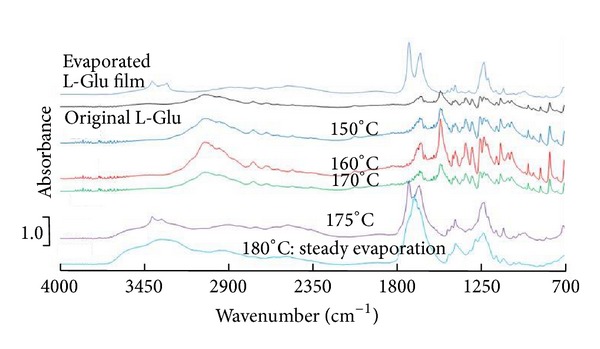
FTIR spectra of the residues in an effusion cell depended on cell temperature.

We have analyzed the residues of the evaporation source L-Glu in the effusion cell to support our analytical discussion on the deposited film. The FTIR spectra of the residues which depended on cell temperature are shown in Figure 9. The IR spectra of the residues heated below 170°C were similar to that of the original L-Glu, whereas they were transformed to that of L-Pygl at 175°C, in which condition the evaporation was considered to virtually start. Below 170°C, the evaporation has not been actually carried out without transforming the molten state. The residues in the effusion cell have retained their original structure in the form of white granules, as charged in the effusion cell.

When the cell temperature reached 175°C, the L-Glu molecules were transformed to the molten L-Pygl responsible for evaporation in the low-vacuum condition. After evaporation at 180°C, the remaining residue was a yellowish hard resin, which was presumably produced by the thermal degradation-polymerization of L-Pygl. Above 1600 cm^−1^, the FTIR spectrum of the residue at 180°C differed from that of L-Pygl, suggesting that the L-Pygl had begun to degrade thermally transforming to hard resin [27]. It is conceivable that the L-Glu-originated Pygl vaporized steadily to form the Pygl films, whereas the polymeric resin was formed as a residue in the effusion cell above 180°C.

**Figure 10:**
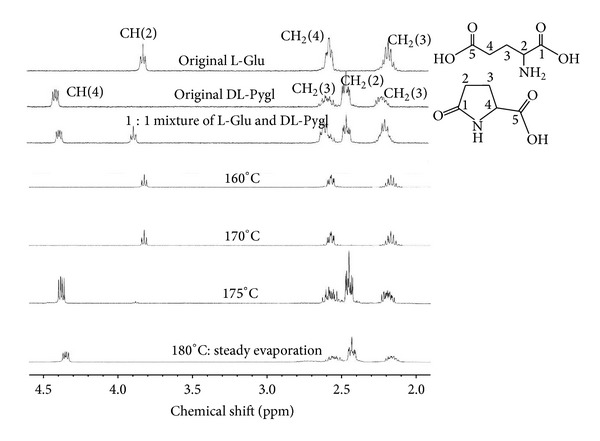
High-middle magnetic field ^1^H-NMR spectra of the residues in an effusion cell depended on cell temperature.

The temperature-dependent ^1^H-NMR spectra of the residues in the effusion cell are shown in Figure 10. These ^1^H-NMR spectra were obtained for the components of residues soluble in deuterium oxide (D_2_O). The residues below 170°C show the essentially same spectra as that of the original L-Glu. The spectra of the residue at 175 and 180°C are similar to that of the 1 : 1 mixture of L-Glu and DL-Pygl, which has been formed by thermal transformation from L-Glu. The remaining residues above 175°C contained the D_2_O-soluble monomers of the pristine L-Glu and the lactamized DL-Pygl, which had not polymerized to be insoluble resin.

### 3.5. Thermodiagram during Evaporation of L-Glu

**Figure 11:**
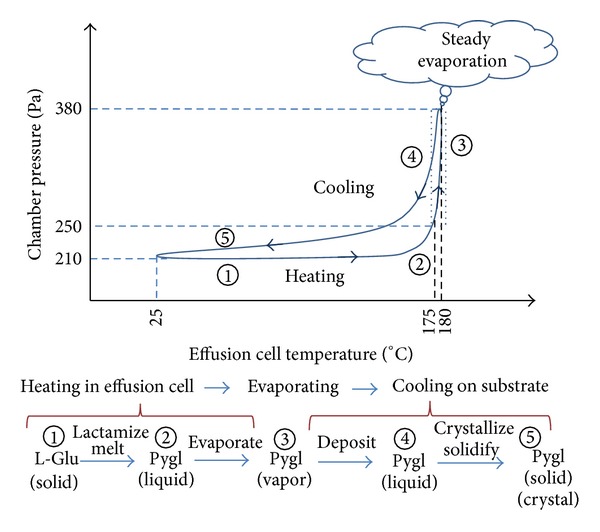
Schematic summary of sequential changes of L-Glu in the course of evaporation-deposition in low-vacuum conditions.

The sequential transformation from original L-Glu in effusion cell to the Pygl film deposited on the substrate in the course of evaporation under low-vacuum condition is schematically summarized in Figure 11. In this scheme, the status diagram of evaporant L-Glu is expressed in the typical correlation curve between the pressure in the evaporation chamber and the temperature of the effusion cell. It is virtually certain that the transformation of L-Glu to DL-Pygl is a critical event for evaporation, leading to the deposition of thin solid films with thermodynamically stable structures.

In this study, we revealed the structures of evaporated L-Glu films by using handmade low-vacuum coater. The evaporated films were mainly composed of L-Pygl and contained slight amounts of racemized DL-Pygl. The applicability of our simple evaporation method under low-vacuum conditions remains to be clarified. We consider amino acids to be an attractive molecular group for forming vaporized films that are suitable for the fabrication of biological devices.

### 3.6. Pressure-Sensing Performance of the QCR Coated with the L-Glu-Evaporated Film

**Figure 12:**
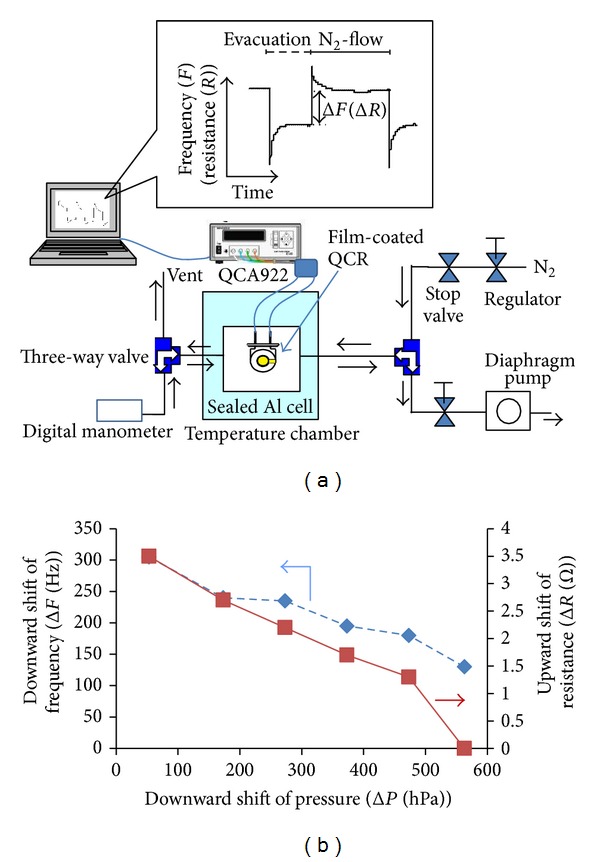
The pressure-sensing performance of the QCR coated with the L-Glu-evaporated film: (a) schematics of the QCR measurement system depending on a change of pressure, (b) the pressure-dependent downward shift of frequency and upward shift of resistance. All of the shifts of frequency, resistance, and pressure are evaluated based on a constant condition under the N_2_-flow (200 mL/min) at 1013 hPa and 25°C.

We have investigated the pressure-sensing performance of the QCR coated with the L-Glu-evaporated film. Varying the pressure in handmade Al cell as shown in Figure 12(a), we have measured the pressure-dependent shifts of frequency (Δ*F*) and of resistance (Δ*R*) from the base condition under the N_2_-flow (200 mL/min) at 1013 hPa and 25°C. As summarized in Figure 12(b), the slight evacuation (small Δ*P*) induces the large downward shift of frequency and the large upward shift of resistance. With increasing downward shift of pressure (Δ*P*) from the base pressure at 1013 hPa, the downward shift of frequency and upward shift of resistance tend to decrease. These tendencies are appropriately explained by the adsorption of residing N_2_ at the surface of the film. The slight evacuation should cause the large amounts of adsorption of residing N_2_, which appeared in the large downward shift of frequency (Δ*F*). The large amounts of N_2_ adsorption should induce the damping effect on resonator by dissipating resonance energy at film surface. This damping effect is ascertained by an increase of resistance (Δ*R*), which corresponded in an equivalent circuit [23, 29–31]. As shown in Figure 12, the QCR coated with the L-Glu-evaporated film has a promising layer for pressure sensing by adsorption-desorption of the surrounding gas.

## 4. Conclusions

The evaporation of L-Glu was investigated for forming thin solid films, using the versatile handmade low-vacuum coater made up of a soldering iron placed in a vacuum-degassing resin chamber. This simple experimental setup is useful for a wide range of organic substances for the evaporation-deposition of organic thin solid films.

The thin solid film evaporated from the molten L-Glu is mainly composed of L-Pygl due to intramolecular lactamization. The structural analyses were conducted with ^1^H-NMR, ^13^C-NMR, FTIR, XRD, DSC, and HPTLC. The major component of the L-Glu-evaporated L-Pygl was in *β*-phase. Additionally, the deposited L-Pygl contained slight amounts of *γ*-phase of L-Pygl, which was presumably generated from the partially racemized DL-Pygl, which is also a minor component of the L-Glu-evaporated film. The DL-Pygl-evaporated film was composed of the original DL-Pygl, retaining its crystal structure. Supported by analyses on the residues in effusion cell, the comprehensive deposition scenario can be presented; namely, L-Glu began to evaporate after the transformation to Pygl, which was evaporable under low-vacuum conditions.

The evaporated L-Glu films generally consisted of 20-nm particulates of Pygl. A periodic single-molecule spacing pattern was conceivable by TEM analysis of the early stage of Pygl deposits. The micromorphology of the deposits, which was basically either domes or plates, was essentially affected by the wettability of the viscous Pygl deposited on the film substrate. The capability of pressure-sensing of the amino acid coated quartz crystal resonator was demonstrated by using the L-Glu-evaporated film.

Our feasible low-vacuum evaporation methodology has potential for preparing thin solid films of evaporable organic materials, which are difficult to form by wet processes.
